# The Impact of the Polymer Chain Length on the Catalytic Activity of Poly(*N*-vinyl-2-pyrrolidone)-supported Gold Nanoclusters

**DOI:** 10.1038/s41598-017-10165-9

**Published:** 2017-08-29

**Authors:** Setsiri Haesuwannakij, Tetsunari Kimura, Yuji Furutani, Kazu Okumura, Ken Kokubo, Takao Sakata, Hidehiro Yasuda, Yumi Yakiyama, Hidehiro Sakurai

**Affiliations:** 1 Department of Functional Molecular Science, School of Physical Science, SOKENDAI (The Graduate University for Advanced Studies), Myodaiji, Okazaki 444-8787 Japan; 2 Institute for Molecular Science, Myodaiji, Okazaki 444-8585 Japan; 3 School of Advanced Engineering. Department of Applied Chemistry, Faculty of Engineering, Kogakuin University, 1-24-2 Nishi-Shinjuku, Shinjuku-ku, Tokyo 163-8677 Japan; 4 Division of Applied Chemistry, Graduate School of Engineering, Osaka University, Suita, Osaka 565-0871 Japan; 5 Research Center for Ultra-High Voltage Electron Microscopy, Osaka University, Ibaraki, Osaka 567-0047 Japan; 6 Division of Materials and Manufacturing Science, Graduate School of Engineering, Osaka University, Suita, Osaka 565-0871 Japan; 7 Department of Chemistry, Graduate School of Science, Present Address: Kobe University 1-1 Rokkodai, Nada-ku, Kobe 657-8501 Japan

## Abstract

Poly(*N*-vinyl-2-pyrrolidone) (PVP) of varying molecular weight (*M*_*w*_ = 40-360 kDa) were employed to stabilize gold nanoclusters of varying size. The resulting Au:PVP clusters were subsequently used as catalysts for a kinetic study on the sized-dependent aerobic oxidation of 1-indanol, which was monitored by time-resolved *in situ* infrared spectroscopy. The obtained results suggest that the catalytic behaviour is intimately correlated to the size of the clusters, which in turn depends on the molecular weight of the PVPs. The highest catalytic activity was observed for clusters with a core size of ~7 nm, and the size of the cluster should increase with the molecular weight of the polymer in order to maintain optimal catalytic activity. Studies on the electronic and colloid structure of these clusters revealed that the negative charge density on the cluster surface also strongly depends on the molecular weight of the stabilizing polymers.

## Introduction

The catalytic activity of gold nanoclusters (AuNCs) strongly depends on their size^1^, whereby smaller clusters usually exhibit higher catalytic activity due to quantum size effects. On account of the charge distribution on the gold surface, the electronic effects are also more advantageous in AuNCs with smaller cores^2^. The catalytic activity of AuNCs is also strongly affected by their interfacial environment. Previous studies have demonstrated that poly(*N*-vinyl-2-pyrrolidone) (PVP)s are well suited to stabilize the surface of AuNCs (Au:PVP)^3^, and that the interactions *via* the carbonyl group do not affect the catalytic activity of these nanoclusters^2, 4^. In general, ligands that strongly coordinate to the AuNC surface, such as thiols, inhibit the catalytic activity of the clusters^5, 6^. However, weakly adsorbing stabilizers such as PVP represent an ideal stabilizing polymer system for AuNCs. In addition to its stabilizing function, PVP may also act as an electron donor, and the coordination of PVP to the gold surface has already been investigated^7–10^.

**Figure 1:**
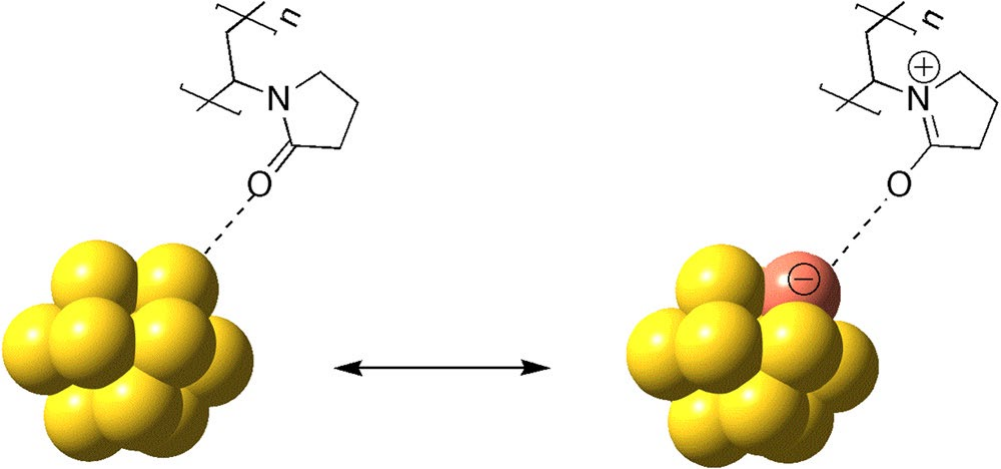
The Interaction between AuNCs and PVP^2, 7, 8^. Mesomeric resonance structures resulting in an electron donation from PVP to the Au surface *via* the C = O group are shown. This electron donation results in the presence of an anionic charge on the Au surface, which promotes the activation of molecular oxygen during the catalytic cycle.

Previous experimental and theoretical studies have shown that the interaction between a stabilizing polymer and AuNCs leads to a donation of negative charges onto the AuNC surface, which is important for catalytic aerobic oxidations involving molecular oxygen (Fig. 1)^11, 12^. The increased electron density on the AuNC surface leads to an enhanced catalytic activity *via* the formation of catalytically active superoxo species, which are generated by an electron transfer from the high-lying LUMO of the anionic AuNC to the π*-orbital of the adsorbed dioxygen molecule^2^.

The fact that the stability of nanoclusters can be controlled by the molecular weight of the stabilizing polymer is well established, whereby polymers with lower molecular weight usually afford less protection against agglomeration^13–15^. Although several reports discuss the stabilization of AuNCs by polymers as a function of the molecular weight, morphology, and rheology^16–19^, studies on the effect of the molecular weight of the polymers on the catalytic activity of the AuNC surface still remain elusive. This is most likely due to the assumption that AuNCs that are stabilized by the same polymer should exhibit comparable activity on account of the comparable environment of the metal surface. We have previously reported a preliminary investigation on the effect of the PVP chain length on the homocoupling of phenylboronic acid. AuNCs stabilized by PVP (K-30; *M*_*W*_ = 40 kDa), *i.e*., Au:PVP (K-30) exhibited higher catalytic activity and selectivity relative to AuNCs stabilized by PVP (K-90; *M*_*W*_ = 360 kDa), *i.e*., Au:PVP (K-90). Nevertheless, it should be noted that on the basis of this report, it is impossible to compare the reactivity between polymers of different chain length, as AuNCs of the same size are required for the suppression of the size effect. In the case of high-viscosity PVP (K-90), AuNCs (1.3 nm) could not be obtained from the conventional batch method^18^. However, the size-controlled synthesis of AuNCs has meanwhile developed further, and a wide range of AuNCs (1-9 nm) stabilized by PVPs of different molecular weight is now available^20, 21^. Therefore, we conducted intensive investigations on the correlation between the catalytic activity of Au:PVP and the PVP chain length. Moreover, we examined the structures of AuNCs in order to determine how these are affected by their interaction with the polymer matrix.

## Results

### Size matters, but ‘smaller’ is not necessarily better than ‘larger’

**Figure 2:**
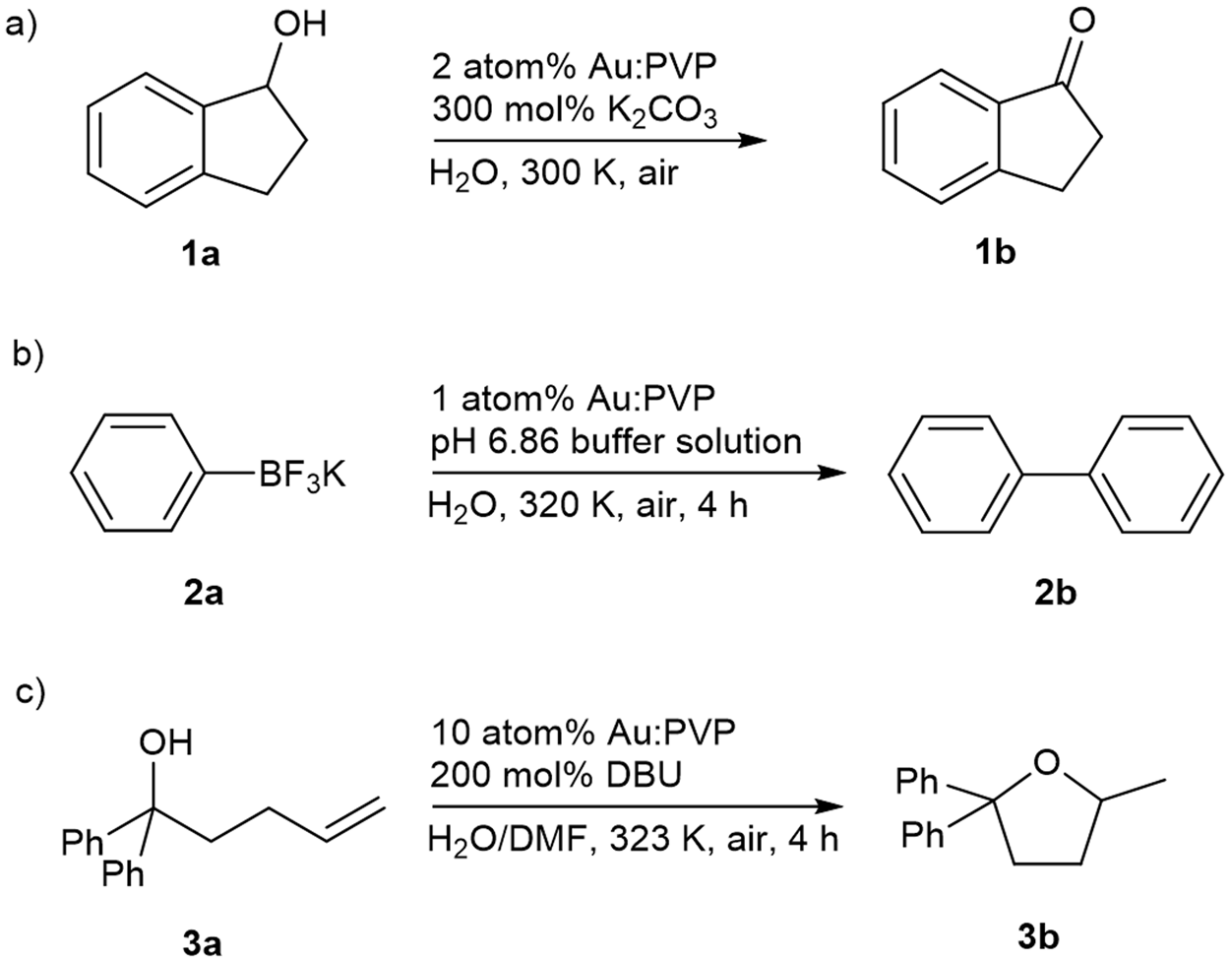
Au:PVP-catalyzed aerobic oxidation reactions. (**a**) aerobic oxidation of 1-indanol. (**b**) aerobic homocoupling of [PhBF_3_]K. (**c**), intramolecular hydroalkoxylation.

**Figure 3:**
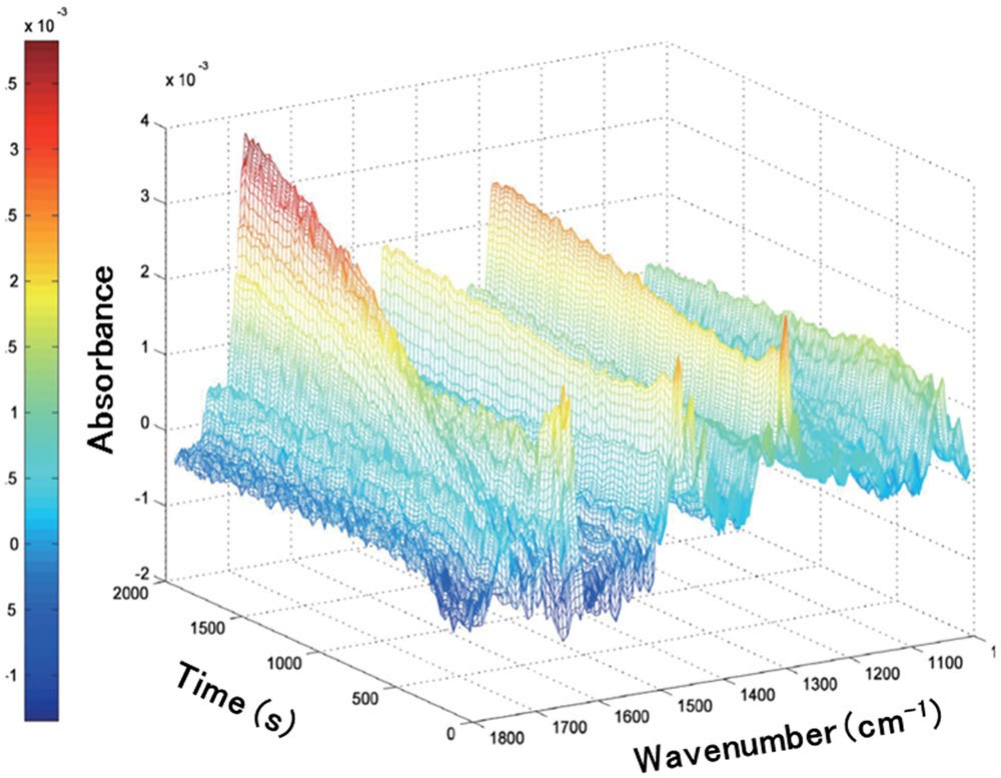
Time-resolved IR spectra of the Au:PVP-catalyzed transformation of 1-indanol to 1-indanone. The reaction profile was monitored using an ATR-FTIR spectrophotometer. The rate of reaction was determined by monitoring the change of the C = O stretching frequency of 1-indanone (**2a**) at 1687 cm^−1^.

AuNCs of varying size, stabilized by PVPs of varying molecular weight, were prepared according to previously reported procedures (Fig. S1–S3 in Supplementary Information)^18, 20, 21^. The size-dependent AuNC-catalyzed oxidation of 1-indanol (Fig. 2a) was selected due to its very short reaction times, the absence of potential alternative pathways, and the possibility to monitor the reaction *in situ* ^22, 23^. This allows avoiding the agglomeration of AuNCs during the reaction, and hence only the catalytic activity of AuNCs is determined. To quantify the matrix effect as well as the size-dependence on the catalytic activity, we examined the aerobic oxidation of 1-indanol (**1a**) catalyzed by AuNCs containing PVP of varying molecular weight, *i.e*., PVP (K-30) (*M*_*W*_ = 40 kDa), PVP (K-60) (*M*_*W*_ = 160 kDa), and PVP (K-90) (*M*_*W*_ = 360 kDa). The progress of this reaction was monitored *in situ* using a fourier transform infrared (FTIR) spectrophotometer with an attenuated total reflection (ATR) accessory unit, which allowed recording the C = O stretching frequency of the reaction product 1-indanone (**2a**). The IR spectra were collected in intervals of 27 seconds, and the obtained time-resolved IR spectra are shown in Fig. 3. The AuNC size and the properties depending on the length of the PVP chain are summarized in Table S1 and Fig. S4 (in Supplementary Information), wherein the normalized rate constant *k*_*norm*_ refers to the reaction rate per unit of surface area which can be obtained by assuming a spherical shape (Supplementary Information)^1, 24^.

**Figure 4:**
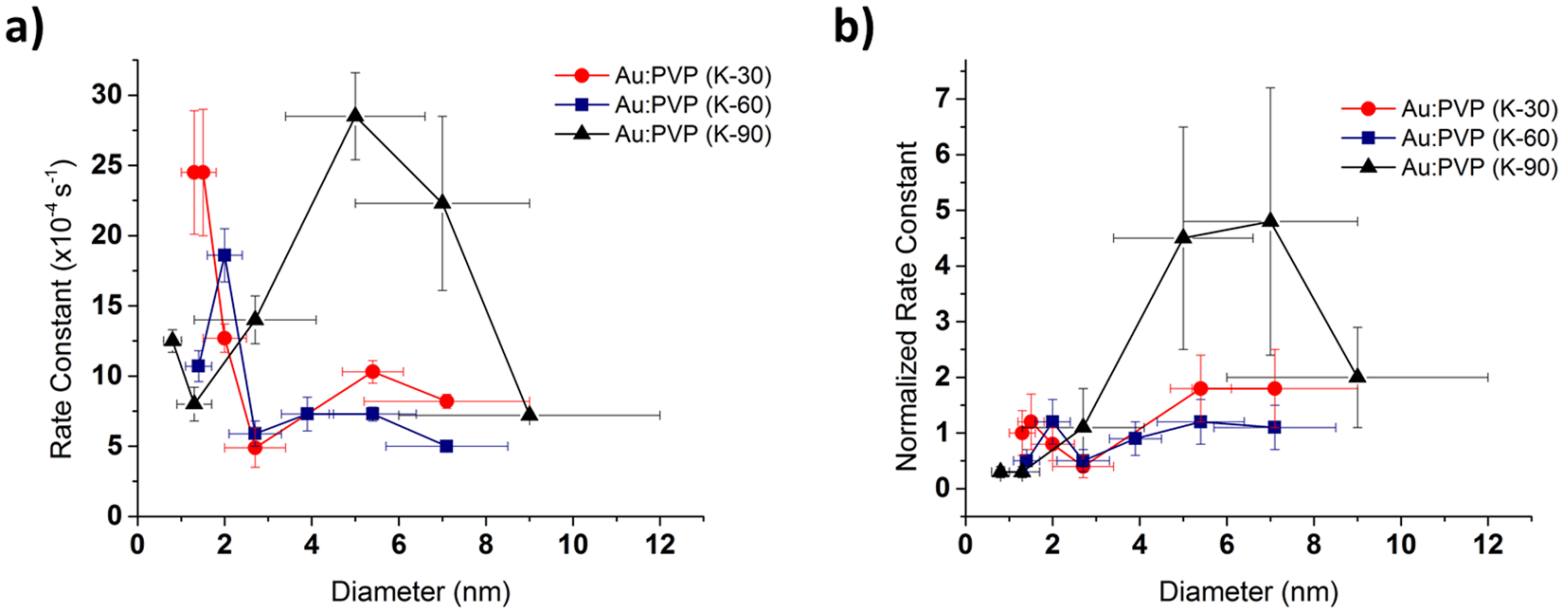
The effect of the PVP chain length and the size of Au:PVP on the catalytic activity in the aerobic oxidation of 1-indanol (**1a**). (**a**) Correlation between the core size and the rate constant. (**b**) correlation between the core size and the normalized rate constant of Au:PVP (K-30) (red), Au:PVP (K-60) (blue), and Au:PVP (K-90) (black). Rate constants were obtained from three independent reactions for each condition and the averaged values with the error bars (the standard deviations) are shown.

The size-dependent properties of Au:PVP (K-30) exhibited a trend similar to that observed in the reaction of *p*-hydroxy benzyl alcohol:^1^ smaller catalysts promote the reaction faster than larger catalyst (Fig. S5 in Supplementary Information). After normalizing the rate constants, the catalytic activity of larger clusters was slightly elevated. In case of Au:PVP (K-60), a similar result was obtained, although the trend was found to shift in favor of larger clusters. However, the size-dependence of Au:PVP (K-90) was substantially different to those of Au:PVP (K-30) and Au:PVP (K-60). For smaller clusters (core size < 2 nm), Au:PVP (K-90) showed inferior activity compared to Au:PVP (K-30), while the activity increased drastically upon increasing the particle size in the range of 0.8–7.0 nm (Fig. 4). The normalized rate constant of Au:PVP (K-90) (core size: 7 nm) was five times higher than that of Au:PVP (K-30) (core size: 1.3 nm), which was previously used as a benchmark catalyst.

Considering the superior catalytic activity of Au:PVP (K-90) (core size: 7 nm) in the catalytic aerobic oxidation of **1a**, we wanted to find out if the observed reactivity is substrate-specific. For that purpose, we examined other typical AuNC-catalyzed reactions, *e.g*. the aerobic homocoupling of organoboron compounds such as potassium trifluorophenyl boronate ([PhBF_3_]K, **2a**)^25^, and the intramolecular hydroalkoxylation of unactivated alkenes such as 1,1-diphenyl-4-penten-1-ol (**3a**)^26, 27^ (Fig. 2b,c).

Subsequently, we investigated the size and matrix effects of these reactions under previously reported conditions^25–27^. The aerobic homocoupling of potassium trifluorophenyl boronate (**2a**) affords biphenyl (**2b**), while the intramolecular hydroalkoxylation of 1,1-diphenyl-4-penten-1-ol (**3a**) furnishes cyclization product **3b**. The approximate activity of each catalyst was determined based on the yield of the corresponding products. The reaction was stopped after 4 h, and the yield of **2b** and **3b** was determined by GC (Table S2 and S3, Fig. S6 in Supplementary Information). In these reactions, similar trends as in the aerobic oxidation of **1a** were observed, *i.e*., the highest catalytic activity was observed for Au:PVP (K-90) (core size: 7 nm). It should also be noted that the reaction time for the aerobic homocoupling, as well as for the intramolecular hydroalkoxylation was substantially shortened from 16–24 h (conventional conditions) to 4 h when Au:PVP (K-90) (core size: 7 nm) was used.

As these results clearly demonstrate that the polymer matrix effect is not limited to specific substrates or reactions, the polymer matrix should play an important role for the catalyst activity. Moreover, these results suggest that it should be possible to tune the catalytic activity of the PVP-stabilized AuNCs via the polymer matrix.

### The electronic structure of Au:PVP

Among the series of Au:PVP derivatives tested, Au:PVP (K-90) (core size: 7 nm) exhibited extremely high catalytic activity in all three reactions. In order to better understand the origins of this outstanding catalytic performance, we wanted to determine the electronic structure of these AuNCs, and find out if an effect of the polymer matrix could be established. The analysis of the electronic structure was achieved by X-ray adsorption (EXAFS and XANES) and X-ray photoelectron spectroscopy (XPS) (Fig. S7–S8 in Supplementary Information).

Table 1 and Table S4 (in Supplementary Information) show the EXAFS and XANES data, whereby the former are in good agreement with the TEM results. The Au-Au coordination number *CN*, as well as the interatomic bond lengths *R* increase with increasing core size. All Au:PVP clusters show a noticeable decrease of bond lengths compared to Au foil (2.88 Å). The contraction of the metallic bond distance in these AuNCs should also lead to changes of their electronic properties^28^. However, the relatively small alteration of the bond lengths in the larger AuNCs relative to Au foil suggests that these should be less effective in catalytic reactions. Moreover, in most of the cases and virtually for all core sizes, Au:PVP (K-90) exhibited significantly smaller coordination numbers than PVP (K-30) and PVP (K-60) (*cf*. entries 1–4, 7–9, 13–15). It can therefore be concluded that the increasing molecular weight of the stabilizing polymer resulted in a morphological change of the metal surface.

**Table 1:** Determination of the Electronic and Colloid Structure of Au:PVP.

Entry	Au:PVP	Core size/nm	*CN*	*R*/Å	Edge energy/eV	%Rf	BE/eV
1	PVP (K-30)	1.3 ± 0.3	6.5	2.787	11916.18^*a*^	1.5	83.75 ± 0.18
2		1.5 ± 0.3	7.0	2.781	11916.06^*a*^	2.3	83.45 ± 0.16
3		2.0 ± 0.5	8.3	2.809	11916.12^*a*^	1.3	83.49 ± 0.16
4		2.7 ± 0.7	8.8	2.817	11916.18^*a*^	0.8	83.51 ± 0.11
5		5.4 ± 0.7	10.4	2.858	11916.13^*a*^	0.4	83.52 ± 0.13
6		7.1 ± 0.7	10.6	2.886	11916.13^*a*^	0.1	83.71 ± 0.04
7	PVP (K-60)	1.4 ± 0.3	7.8	2.810	11916.12^*a*^	1.4	83.77 ± 0.27
8		2.0 ± 0.4	8.0	2.826	11916.18^*a*^	1.4	83.25 ± 0.09
9		2.7 ± 0.6	8.4	2.829	11916.12^*a*^	2.4	83.65 ± 0.24
10		3.9 ± 0.6	8.5	2.858	11916.13^*a*^	0.5	83.67 ± 0.33
11		5.3 ± 0.7	10.5	2.860	11916.07^*a*^	0.3	83.87 ± 0.04
12		7.2 ± 0.7	10.7	2.876	11916.08^*a*^	0.3	83.97 ± 0.02
13	PVP (K-90)	0.8 ± 0.2	6.0	2.773	11920.14^*b*^	2.0	83.78 ± 0.10
14		1.3 ± 0.4	6.0	2.773	11919.88^*b*^	1.6	83.80 ± 0.28
15		2.7 ± 1.4	8.0	2.786	11920.06^*b*^	0.6	83.78 ± 0.23
16		5.0 ± 1.6	9.0	2.810	11916.03^*a*^	0.8	83.39 ± 0.10
17		7.0 ± 2.0	9.1	2.805	11916.08^*a*^	2.3	82.62 ± 0.10
18		9.1 ± 3.4	10.6	2.817	11920.0^*b*^	0.9	84.10 ± 0.14

*CN* = coordination number, *R* = interatomic bond distance, BE = binding energy; ^a,b^The edge energy of Au foil was 11916.3 and 11920.3 eV, respectively.

Although an analysis of the XANES-derived edge energy values for the Au:PVP clusters suggested that their Au cores exhibit a more anionic character than Au foil, it should be noted that the comparison of the observed small edge energy differences was difficult, mostly due to the low resolution and concentration of gold in these clusters system. Therefore, we decided to further examine the size-dependence of the electronic structures of the Au:PVP clusters by XPS.

The electron density of the Au cores can be determined experimentally by XPS *via* the binding energy (BE) of Au4*f*_7/2_. The XPS data showed a primary Au4*f*_7/2_ band for the Au atoms accompanied by the corresponding satellite peaks. The width of the observed peaks is thereby indicative of a contribution of Au atoms with different electron density. Particularly, the significantly smaller BE of Au4*f*_7/2_ relative to bulk gold (84.0 eV) suggests that the negative charge on the gold surface in Au:PVP arises from the interaction between the gold surface and the polymer matrix (Table 1, Fig. S8 in Supplementary Information)^2^. The BE of Au:PVP (K-90) (entries 13–17) decreases with increasing core size (≤ 9 nm) before increasing with further increasing core size. Au:PVP (K-90) (core size: 9 nm) (entry 18) exhibits a significantly increased BE relative to the smaller clusters, due to the decreased electron density on the gold surface. The Au 4*f*_7/2_ BE for Au:PVP (K-90) (core size: 7 nm) (entry 17) is clearly smaller than that for the other clusters, indicating the highest negative charge on its surface, which is in good agreement with the results of the catalytic activity study.

**Figure 5:**
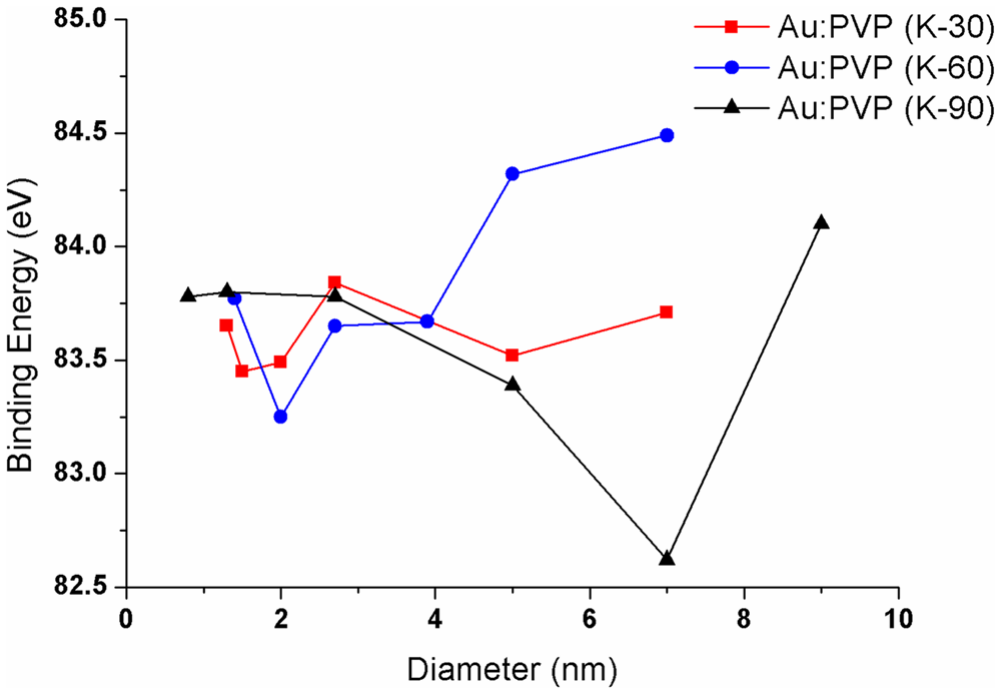
Correlation between the BE of Au4*f*_7/2_ and the core size of Au:PVP. Au:PVP (K-30) (red), Au:PVP (K-60) (blue), and Au:PVP (K-90) (black).

Although the BE of Au4*f*_7/2_ and its catalytic activity are not perfectly proportional (Fig. 5), it should nevertheless be useful to compare these correlations with similar catalyst systems, *e.g*., with Au:PVP (core size: 1.3 nm). The smallest BE value was observed for Au:PVP (K-30) (entry 1), followed by Au:PVP (K-60) (entry 7), and Au:PVP (K-90) (entry 14). These results are consistent with the experimental data that Au:PVP (K-30) with small cores exhibited the highest catalytic activity (Fig. 4a).

Another possible explanation is the geometrical effect by changing the crystallinity. For example, the TiO_2_-supported poly-crystalline Au showed twice the activity of that single crystalline Au/TiO_2_ system^29^. Tsukuda and co-workers also reported that icosahedral Au_144_ clusters supported on hierarchically porous carbon exhibited superior catalytic activity to the smaller fcc Au clusters^30^. To confirm the structural differences in each clusters, we carried out the high-resolution TEM (HR-TEM) and powder X-ray diffraction (PXRD) measurements. HR-TEM measurement revealed that, in case of relatively larger cluster (more than 5 nm), most of the cores were polycrystals regardless to the polymer length (Fig. S2, Supplementary Information). In addition, PXRD measurement suggested that the larger clusters mainly keep fcc structures (Fig. S3, Supplementary Information)^31^. Although this morphological effect on the catalytic activity should not be ignored, it is difficult to explain the significant difference of the catalytic activity of 7 nm-sized Au:PVP (K-30) and Au:PVP (K-90). Therefore, AuNCs should thus be intimately correlated with mainly the electronic properties on the surface of the gold cluster, and it can hence be concluded that the excellent catalytic activity of Au:PVP (K-90) (core size: 7 nm) may be attributed to the highly negative charge density on its surface.

The electronic structure studies also revealed that Au:PVP (K-90) (core size: 7 nm), which exhibited high reactivity, possessed more anionic nature. This behavior may be rationalized in terms of electron affinity on the gold surface, as the partial electron transfer from PVP to the core gold leads to bond activation of the substrate prior to participation in the catalytic cycle^25^.

### Structural analysis of the colloidal Au:PVP

**Figure 6:**
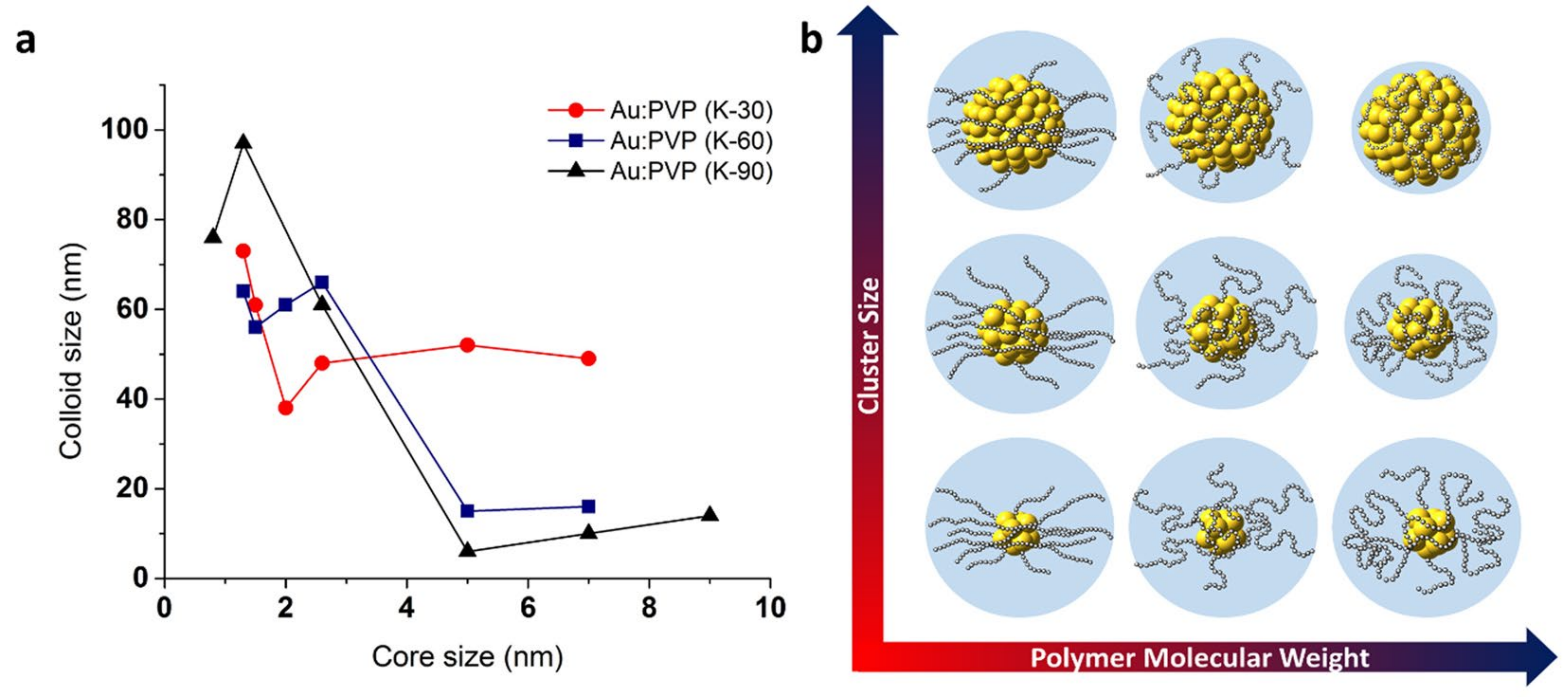
Colloid size of Au:PVP determined by the IG method. The data shows the relationship between colloid size and core size of AuNCs stabilized by PVPs of varying molecular weight. (**a**) Au:PVP (K-30) (red), Au:PVP (K-60) (blue), and Au:PVP (K-90) (black). Entangled structures are expected upon matching long-chain polymers and large metal cores; (**b**) effect of the molecular weight of the polymers and the cluster size on the colloid structure.

Considering the aforementioned results in their entirety, it can be concluded that the excellent catalytic activity of Au:PVP (K-90) (core size: 7 nm) should be ascribed to the high negative charge on the cluster surface. This however poses the question: why does Au:PVP (K-90) (core size: 7 nm) possess such a highly electronegative surface? In order to answer this question, we examined the physical structure of the AuNCs and the effect of the stabilizing PVP on the AuNCs surface. We measured the size of the Au:PVP colloids by the induced grating method (IG method), which includes an activation procedure induced by dielectrophoresis to form a particle grating and measures the decay of the diffracted light. It thus provides a highly sensitive and reproducible means to measure single colloids down to the nanometer scale. The advantage of the IG method over dynamic light scattering (DLS) is that the former is immune to the inaccuracies arising from contamination with large particles, with which the results of the latter are inevitably associated^32, 33^. Fig. 6 and Table S5 (in Supplementary Information) show the relationship between Au:PVP colloid size and core size. The colloid size of most Au:PVP (K-30) was 40 ± 10 nm, irrespective of the core size and the polymer chain length, while the colloid size of free PVP was ~100–200 nm. The colloid size of both Au:PVP (K-60) and Au:PVP (K-90) decrease with increasing core size (Fig. S9–S10 in Supplementary Information).

**Figure 7:**
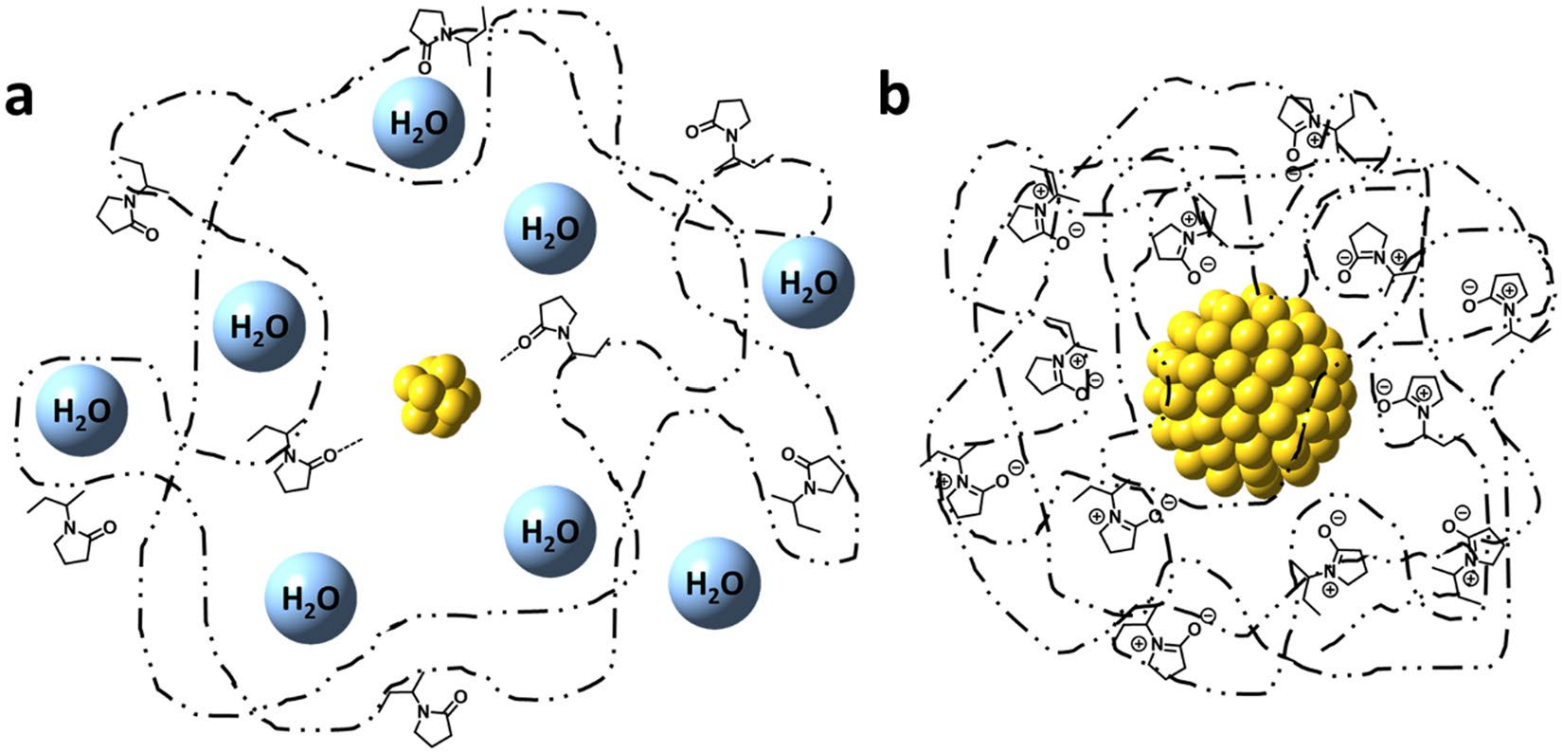
Effect of the polymer chain length on the colloid structure of Au:PVP. (**a**) Small clusters stabilized by short-chain polymers incorporate water molecules in loose colloid structures; (**b**) larger clusters stabilized by long-chain polymers afford highly entangled structures due to the stronger surface interaction between PVP and the metal core.

Judging from the volume in the presence of short-chained polymers, Au:PVP colloids consist predominantly of water (>90%), indicating that the gold cluster should be drifting in diluted aqueous PVP solutions, reminiscent to an egg of frog model (Fig. 7a), which reminds us a frog egg surrounded by jelly-like blob in frogspawn. Recent results obtained from MD-DFT calculations indicated that PVP moieties bind much closer to metal surfaces than H_2_O. Consequently, the PVP concentration should be increased in close spatial proximity of the cluster, which might explain the stabilizing effect of PVP^31^. However, the physical structure of the interface between the gold surface and the PVP matrix must be loose and soft. In contrast, when the bigger clusters are wrapped with high-molecular-weight polymers, the polymer should wrap around the Au core and thus afford higher surface coverage^34^ and more entangled structures (Fig. 7b). In such entangled structures, the hydrogen-bonding network should be eliminated, which would result in contracted colloids. Indeed, the aforementioned calculations suggested that virtually no water should be included in Au:PVP (K-90) (core size: 7 nm) colloids. Therefore, it can be concluded that a strong interfacial interaction is induced when the chain length of the stabilizing polymer and the core size of the metal cluster are matched, emerging the highly electronegative surface on the metal core.

## Conclusion

In summary, we were able to demonstrate that Au:PVP catalysts promote a variety of reactions. In all cases, the properties of Au:PVP depend on the cluster size. However, morphological effects can surpass the size effect and control the catalytic activity. From an applications-driven perspective, it is highly promising that the catalytic activity of AuNCs can be modulated by the cluster size and by the polymer matrix. Our experimental results revealed that Au:PVP (K-90) (core size: 7 nm) exhibited a catalytic activity that was up to five times higher than that of Au:PVP (K-30) (core size: 1.3 nm). The extremely high activity of the former should be ascribed to the high surface coverage of the gold core with PVP (K-90), which should lead to a high density of negative charges on the core surface. The analysis of the physical structure suggested a difference in surface morphology. Even though the interface between the Au surface and the PVP matrix is usually loose, this is not the case for long PVP chains. AuNCs stabilized by long-chain polymers result in highly entangled structures *via* entanglement of the Au surface with PVP, which increases the electronegativity on the surface of the cluster. However, it is still extremely difficult to experimentally determine the structure of this interface precisely, and new experimental methods as well as the computational studies are required to advance research in this area.

## Electronic supplementary material

supporting information
