# Supplementary material for: The Impact of the Polymer Chain Length on the Catalytic Activity of Poly(N-vinyl-2-pyrrolidone)-supported Gold Nanoclusters
Source: Sci Rep. 2017 Aug 29;7:9579. doi: 10.1038/s41598-017-10165-9 (PMC5575105; doi:10.1038/s41598-017-10165-9)
Supplement: Supplementary file 1 — supporting information [file 41598_2017_10165_MOESM1_ESM.pdf]

Supplementary Information

The Impact of the Polymer Chain Length on the Catalytic Activity of Poly(*N*-vinyl-2-pyrrolidone)-supported Gold Nanoclusters

Setsiri Haesuwannakij[1], Tetsunari Kimura[1,2,#], Yuji Furutani[1,2], Kazu Okumura[3], Ken Kokubo[4] Takao Sakata[5], Hidehiro Yasuda[5,6], Yumi Yakiyama[4] & Hidehiro Sakurai[4]

[1]Department of Functional Molecular Science, School of Physical Science, SOKENDAI (The Graduate University for Advanced Studies), Myodaiji, Okazaki 444-8787, Japan.

[2]Institute for Molecular Science, Myodaiji, Okazaki 444-8585, Japan.

[3]School of Advanced Engineering. Department of Applied Chemistry, Faculty of Engineering, Kogakuin University, 1-24-2 Nishi-Shinjuku, Shinjuku-ku, Tokyo 163-8677, Japan

[4]Division of Applied Chemistry, Graduate School of Engineering, Osaka University, Suita, Osaka 565-0871, Japan

[5]Research Center for Ultra-High Voltage Electron Microscopy, Osaka University, Ibaraki, Osaka 567-0047, Japan

[6]Division of Materials and Manufacturing Science, Graduate School of Engineering, Osaka University, Suita, Osaka 565-0871, Japan

Experimental Section

1: General

High-resolution TEM images were recorded with a JEOL JEM-2100F and HITACHI HF-2000 at an accelerating voltage of 200 kV. UV-vis spectra were measured using a spectrophotometer (JASCO V-670) at 25°C. X-ray photoelectron spectroscopy (XPS) of Au:PVP was conducted using a Vacuum Generators ESCALAB 220iXL spectrometer. The most intense peak for C1s which obtain from PVP was set at 284.6 eV as an internal standard. Powder X-ray diffraction measurement was carried out using Rigaku RINT-2000 using graphite-monochromitized Cu-Kα radiation (λ = 1.54187 Å) at room temperature. The single nanoparticle size analyzer: induced grating method (IG method) was carried out using Shimadzu IG-1000 Plus: Single Nano Particle Size Analyzer. The measurement was

carried out under 25 °C with frequency of 1 kHz and 25 V of applied voltage. The kinetic of aerobic oxidation of 1-indanol was in situ observed by FT-IR spectrophotometer, Bruker Vertex 70 FT-IR spectrometer with attenuated total reflectance (ATR) device at 27°C. Gas chromatography 2010 (Shimadzu) with Rtx-5MS column length 30m, inner diameter 0.25 mm and film thickness 0.25 µm was used for determination of the yield.

All chemicals and solvents were used as received without further purification unless otherwise noticed. Hydrogen tetrachloroaurate tetrahydrate ($HAuCl_4.4H_2O$ Tanaka Kikinzoku) and sodium tetraborohydride ($NaBH_4$, Wako) and poly(*N*-vinyl-2-pyrrolidone) (Kishida chemicals) were used as precursors for the preparation of gold nanoclusters. Ethyl acetate was purchased from Wako. 1-indanol was purchased from TCI. Potassium hydroxide, potassium carbonate and DMF were obtained from Wako. Milli-Q grade water was used in all experiments.

2: Sample preparation

Preparation of polymer-stabilized gold nanoclusters by rapid reduction by $NaBH_4$

The rapid reduction was followed the reported procedure.[1] Rapid reduction of $HAuCl_4$ by $NaBH_4$ was performed in an aqueous solution of PVP under 0°C to yield a brown hydrosol of Au:PVP. The molar ratio of $HAuCl_4$, monomer unit of PVP, and $NaBH_4$ was maintained at 1:100:10. The resulting solution mixture was concentrated to 10 mL by centrifugal ultrafiltration at 4800 rpm by using a membrane with an appropriate molecular weight cut off and washed with pure water three times. The purified AuNC was stored in the solid form in a dry place after freeze-drying with a Freeze Dryer (EYELA FDU-2200).

Preparation of Polymers-stabilized Gold Nanoclusters by Micro-flow technique

The preparation was followed the reported procedure.[2] The molar ratio of tetrachloroauric acid ($HAuCl_4$): polymers: sodium tetraborohydride ($NaBH_4$) was set as 1:100:10, which was the same ratio as the standard batch process. Two aqueous solutions were prepared: one (solution A) is the mixed solution of $HAuCl_4$ and polymers (0.025 mmol for $HAuCl_4$ and 2.5 mmol for PVP (K-90; M_w = 360 kDa), respectively) in 25 mL of water, and another one (solution B) is a solution of $NaBH_4$ (0.25 mmol of $NaBH_4$) in 5 mL of water. Solutions, micro-reactor, flow channel, and the receiver were kept cooling at 0 °C. The flowrate was controlled by syringe pump. The resulting colloidal nanogold was dialyzed three times to remove the inorganic impurities such Na^+ and Cl^- by centrifugal ultrafiltration at 4800 rpm by using a membrane with a molecular weight cut off of 100 kDa using 10ml of water. The resulting solution mixture was concentrated to 10 mL. The purified nanogold was kept as a solid form in a dry place after freeze-drying by using Freeze Dryer (EYELA FDU-2200).

Preparation of polymer-stabilized gold nanoclusters by slow reduction method

The slow reduction was followed the reported procedure.[3] Slow reduction of $HAuCl_4$ was done in a weak basic condition. To a 40 mL aqueous solution containing 0.025 mmol of $HAuCl_4$, PVP (277.8 mg, 2.5 mmol) was added. The mixture was stirred at 0 °C for 5 min. A freshly prepared solution of Na_2SO_3 (9.45 mg, 0.075 mmol) in 5 mL pure water was rapidly added to the mixture, which was stirred vigorously at 1,700 rpm for 3 min. Then, 5 mL of $NaBH_4$ (9.46 mg, 0.25 mmol) was rapidly added to the mixture to yield a brown hydrosol of Au:PVP (K-15). The mixture was stirred vigorously at 1700 rpm for 3 h at 0°C. The resulting solution mixture was concentrated to 10 mL by centrifugal

ultrafiltration at 4800 rpm by using a membrane with an appropriate molecular weight cut off and washed with pure water three times. The purified AuNC was stored in the solid form in a dry place after freeze-drying with a Freeze Dryer (EYELA FDU-2200).

Preparation of polymer-stabilized gold nanoclusters by seed-mediated growth method of Au:PVP

Seed Au:PVP was prepared by rapid reduction of $HAuCl_4$ by $NaBH_4$ in an aqueous solution of PVP under 0°C. This process yielded a brown hydrosol of seed Au:PVP. The molar ratio of $HAuCl_4$ and the monomer unit of PVP was maintained at 1:100, following the reported procedure.[3,4]

To the mixture of degassed aqueous solution of $HAuCl_4$, PVP, and seed Au:PVP, 300 mol% of degassed 90 mM of Na_2SO_3 was added quickly. The solution was stirred continuously at 27°C for 3 h under N_2 atmosphere. The resulting solution was concentrated to 10 mL by centrifugal ultrafiltration at 4800 rpm by using a membrane with an appropriate molecular weight cut off and washed with pure water three times. The purified AuNC was stored in the solid form in a dry place after freeze-drying with a Freeze Dryer (EYELA FDU-2200).

3: General procedure for catalytic oxidation reactions

General procedure for aerobic oxidation of 1-indanol catalyzed by Au:PVP

The 50 μL aqueous solution of 0.67 mM Au:PVP (K-90) was added to 50 μL aqueous solution of 1-indanol 1.67 μmol, K_2CO_3 5 μmol at 27 °C. To prevent the effect from the aggregation of nanogold, the very short reaction time as aerobic oxidation of indanol was selected as a model reaction.

The reaction was in situ observed by FT-IR spectrophotometer with attenuated total reflectance (ATR) method to monitor the absorbance change at the C=O stretching frequency (1687 cm^{-1}) of indanone which is product of this reaction. The infrared spectra were collected at 27 second intervals.

The rate constants were determined by following pseudo-first order reaction rate law of the production of oxidized product. The rate constants were normalized with AuNC's effective surface area by using the values of 1.3 nm Au:PVP (K-30) as standard as follows.[4,5]

$$k_{norm} = \frac{d_n}{d_{ref}} \times \frac{k_n}{k_{nref}}$$

where d_n: the core diameters and k_n: rate constant (before normalization). For the reference value d_{ref} and k_{nref}, we used the data of Au:PVP (K-30) with the size of 1.3±0.3 nm (d_{ref} = 1.3 nm, k_{nref} = 24.5 × 10^{-4} s^{-1})."

General procedure for aerobic homocoupling of potassium phenyltrifluoroborates catalyzed by Au:PVP[6]

Potassium phenyltrifluoroborates ($PhBF_3K$) were prepared according to the literature procedure.[7] Aerobic oxidative homocoupling of potassium phenyltrifluoroborates was carried out using an organic synthesizer (EYELA, PPS-2510). $PhBF_3K$ (0.15 mmol) and 1.8 mL of pH 6.86 phosphate buffer (Wako Pure Chemical Industries) was placed in a test tube (Φ = 15 mm). The aqueous solution of Au:PVP (0.5 mM, 3 mL, 1 atom%) was added and the reaction mixture was stirred (1300 ± 5 rpm) at a given temperature. The reaction was quenched by adding 5 mL of EtOAc and the aqueous phase was extracted three times with 10 mL of EtOAc. The combined organic phase was dried over Na_2SO_4, and evaporated in vacuo. The products were characterized by ^1H NMR. If necessary, further purification was performed by PTLC.

The normalized activity x_{norm} was assumed with AuNC's effective surface area by using the values of 1.3 nm Au:PVP (K-30) as standard as follows.[4,5]

$$x_{norm} = \frac{d_n}{d_{ref}} \times \frac{x_n}{x_{nref}}$$

where d_n: the core diameters and x_n: reaction yield (before normalization). For the reference value d_{ref} and x_{nref}, we used the data of Au:PVP (K-30) with the size of 1.3±0.3 nm (d_{ref} = 1.3 nm, x_{nref} = 21%).

General procedure for intramolecular hydroalkoxylation of 1,1-diphenyl-4-penten-1-ol catalyzed by Au:PVP[8,9]

The 1,1-diphenyl-4-penten-1-ol was prepared according to our previous report.[8] Intramolecular hydroalkoxylation of 1,1-diphenyl-4-penten-1-ol was carried out using an organic synthesizer (EYELA, PPS-2510). 0.03 mmol of substrate and 200 mo% DBU in 3 mL of DMF were placed in a test tube (Φ = 30 mm). A solution of Au:PVP (0.5 mM, 6 mL, 10 atom%) was added and the mixture was stirred vigorously (1300 rpm) at 50 °C for the required time. The reaction was then quenched with 1M HCl and the products were extracted methyl tert-butyl ether with (4 x 15 mL). The extracted organic layer was dried (Na_2SO_4) and concentrated in vacuo. The yield was determined by using gas chromatography; the yield was obtained from the calibration curve.

The normalized activity x_{norm} was assumed with AuNC's effective surface area by using the values of 1.3 nm Au:PVP (K-30) as standard as follows.[4,5]

$$x_{norm} = \frac{d_n}{d_{ref}} \times \frac{x_n}{x_{nref}}$$

where d_n: the core diameters and x_n: reaction yield (before normalization). For the reference value d_{ref} and x_{nref}, we used the data of Au:PVP (K-30) with the size of 1.3±0.3 nm (d_{ref} = 1.3 nm, x_{nref} = 22%).

4: Detailed procedure for sample evaluation

XAS data analysis

A Si (111) single crystal was used to obtain a monochromatic X-ray beam. Measurement was recorded in the quick mode at room temperature. The data were analyzed using the REX2000, ver. 2.5.9, program (Rigaku Co.). Fourier transform of $k3\chi(k)$ data was performed in k ranges of 30-129 nm^{-1} for analysis of the Au L_{III}-edge EXAFS spectra. Inversely Fourier-filtered data were analyzed using the common curve-fitting method. Phase shift and amplitude for Au-Au were extracted from the data by using the FEFF code (ver. 8) with an Au foil. The data were collected in the transmission mode using ion-chamber detectors.

Characterization by transmission electron microscopy

The AuNC solution was purified and concentrated by centrifugation at 4,800 rpm. A drop of the concentrated solution was placed in the copper grid, pretreated with hydrophilic treatment, and then vacuum-dried before measurement. Histograms of size distribution were obtained by measuring the core diameter more than 300 particles. A Gaussian distribution was used to determine core size distribution.

5: Supplementary figures and tables

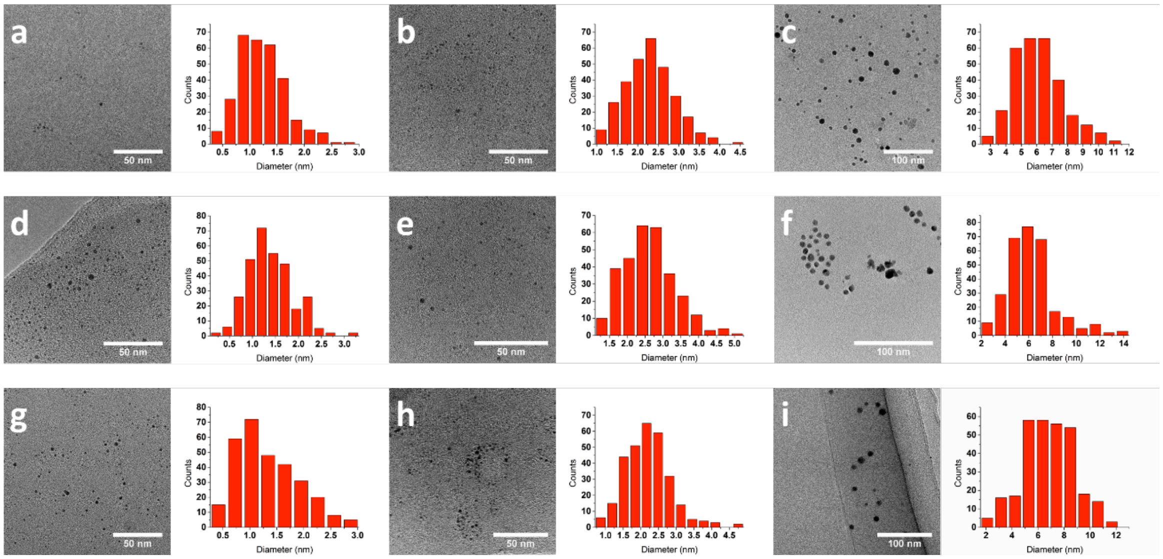

Figure S1. TEM images and core size distribution of Au:PVP. a-c, Au:PVP (K-30) with the core size of 1.3±0.3 nm, 2.7±0.7 nm and 7.1±0.7 nm, respectively. **d-f**, Au:PVP (K-60) with the core size of 1.4±0.3 nm, 2.7±0.6 nm and 7.2±0.7 nm, respectively. **g-i**, Au:PVP (K-90) with the core size of 1.3±0.4 nm, 2.7±1.4 nm and 7.0±2.0 nm, respectively.

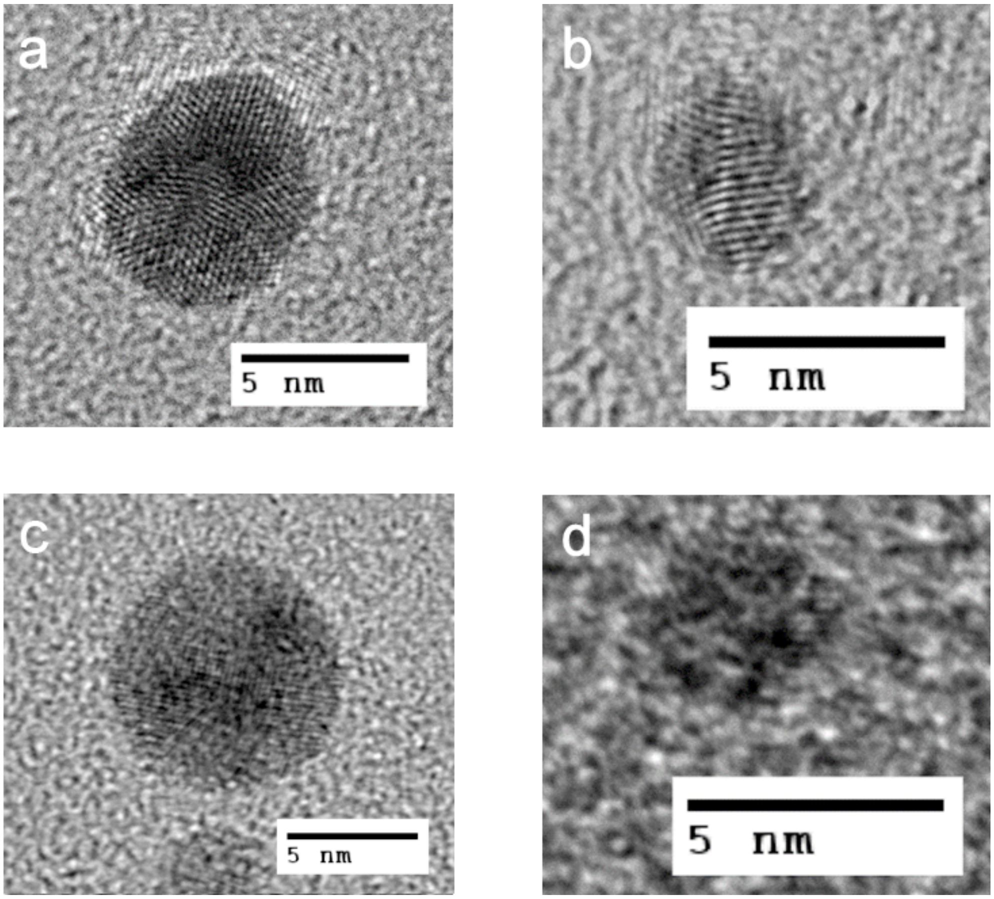

Figure S2. HR-TEM images of Au:PVPs. **a**, Au:PVP (K-30) with the core size of 7.1±1.9 nm. **b**, Au:PVP (K-30) with the core size of 1.3±0.3 nm. **c**, Au:PVP (K-90) with the core size of 7.2±2.0 nm. **d**, Au:PVP (K-90) with the core size of 1.3±0.4 nm. Because of the machine limitation, **b** and **d** show the smallest image which we could observed.

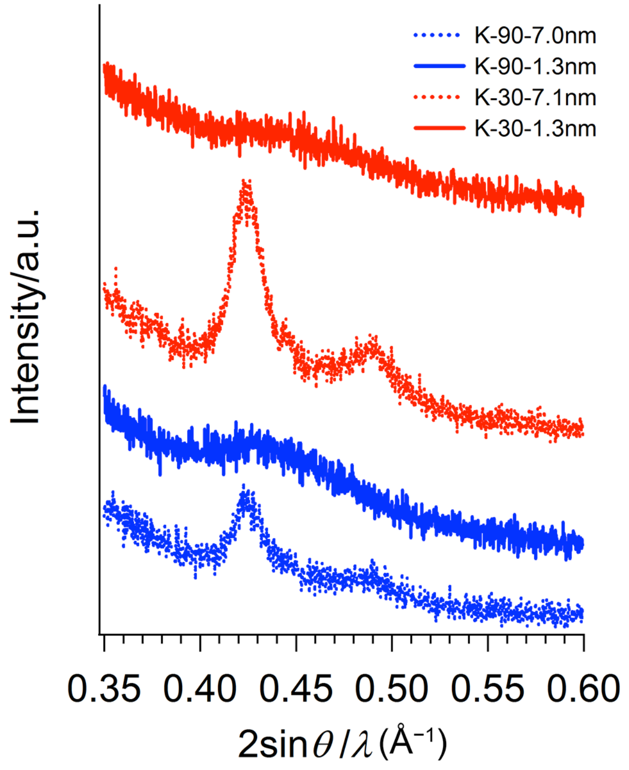

Figure S3. PXRD patterns of Au:PVPs. From Scherrer equation, the estimated smallest particle size of 7 nm Au:PVPs were 4.8 nm (for PVP (K-30)); 4.5 nm (for PVP (K-90)), respectively. These values were corresponding to the size distributions which were obtained by TEM measurements (Figure S1).

Here Scherrer equation is given,

$$D = K\lambda/\beta\cos\theta$$

where D is the mean size of the ordered (crystalline) domains; K is a dimensionless shape factor (here 0.89), λ is X-ray wavelength, β is the line broadening at half the maximum intensity (in radians). θ is the Bragg angle (in degrees).

Table S1. Catalytic activity toward aerobic oxidation of 1-indanol catalyzed by Au:PVP

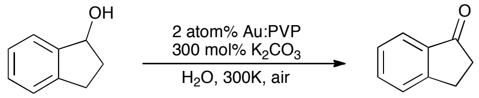

1a **1b**

Entry	PVP	Core size (nm)	Rate Constant (1b)	
			(x 10^{-4} s^{-1})	Normalized rate constant, k_{norm}[a]
1	K-30	1.3±0.3	24.5±4.4	1.0±0.4
2		1.5±0.3	24.5±4.5	1.2±0.5
3		2.0±0.5	12.7±1.0	0.8±0.3
4		2.7±0.7	4.9±1.4	0.4±0.2
5		5.4±0.7	10.3±0.8	1.8±0.6
6		7.1±1.9	8.2±0.5	1.8±0.7
7	K-60	1.4±0.3	10.7±1.1	0.5±0.2
8		2.0±0.4	18.6±1.9	1.2±0.4
9		2.7±0.6	5.9±0.9	0.5±0.2
10		3.9±0.6	7.3±1.2	0.9±0.3
11		5.3±1.0	7.3±0.5	1.2±0.4
12		7.2±1.4	5.0±0.3	1.1±0.4
13	K-90	0.8±0.2	12.5±0.8	0.3±0.1
14		1.3±0.4	8.0±1.2	0.3±0.1
15		2.7±1.4	14.0±1.7	1.2±0.7
16		5.0±1.6	28.5±3.1	4.5±2.0
17		7.0±2.0	22.3±6.2	4.9±2.4
18		9.0±3.0	7.2±0.2	2.0±0.9

[a]$k_{norm} = d_n/d_{ref} \times k_n/k_{nref}$, where d_n: the core diameters and k_n: rate constant (before normalization). For the reference value d_{ref} and k_{nref}, we used the data of Au:PVP (K-30) with the size of 1.3±0.3 nm (d_{ref} = 1.3 nm, k_{nref} = 24.5 × 10^{-4} s^{-1}).

Table S2. Catalytic activity toward homocoupling of potassium phenyltrifluoroboronate by Au:PVP

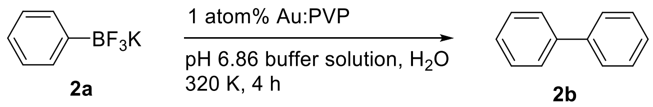

Entry	PVP	Core size (nm)	2b	
			Yield(%)	Normalized activity, x_{norm}[a]
1	K-30	1.3±0.3	21	1.0
2		1.5±0.3	30	1.6
3		2.0±0.5	33	2.4
4		2.7±0.7	32	3.1
5		5.4±0.7	8	2.0
6		7.1±1.9	3	1.0
7	K-60	1.4±0.3	9	0.5
8		2.0±0.4	30	2.2
9		2.7±0.6	24	2.3
10		3.9±0.6	10	1.4
11		5.3±1.0	12	3.0
12		7.2±1.4	13	4.4
13	K-90	0.8±0.2	6	0.2
14		1.3±0.4	8	0.4
15		2.7±1.4	12	1.2
16		5.0±1.6	20	3.7
17		7.0±2.0	99	25.3
18		9.0±3.0	67	22.0

[a] $x_{norm} = d_n/d_{ref} \times x_n/x_{nref}$, where d_n: the core diameters and x_n: reaction yield (before normalization). For the reference value d_{ref} and x_{nref}, we used the data of Au:PVP (K-30) with the size of 1.3±0.3 nm (d_{ref} = 1.3 nm, x_{nref} = 21%).

Table S3 Catalytic activity toward hydroalkoxylation of 1,1-diphenyl-4-penten-1-ol catalyzed by Au:PVP.

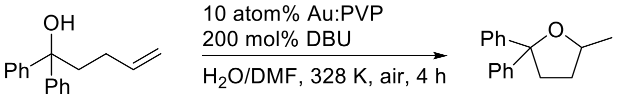

3a **3b**

Entry	PVP	Core size (nm)	3b	
			Yield(%)	Normalized activity, x_{norm}[a]
1	K-30	1.3±0.3	22	1.0
2		1.5±0.3	31	1.6
3		2.0±0.5	36	2.5
4		2.7±0.7	1	0.1
5		5.4±0.7	3	0.6
6		7.1±1.9	4	1.0
7	K-60	1.4±0.3	72	3.5
8		2.0±0.4	55	3.8
9		2.7±0.6	63	5.9
10		3.9±0.6	2	0.4
11		5.3±1.0	14	2.6
12		7.2±1.4	7	1.8
13	K-90	0.8±0.2	77	2.1
14		1.3±0.4	61	2.7
15		2.7±1.4	75	7.1
16		5.0±1.6	83	14.5
17		7.0±2.0	83	20.3
18		9.0±3.0	59	18.5

[a]$x_{norm} = d_n/d_{ref} \times x_n/x_{nref}$, where d_n: the core diameters and x_n: reaction yield (before normalization). For the reference value d_{ref} and x_{nref}, we used the data of Au:PVP (K-30) with the size of 1.3±0.3 nm (d_{ref} = 1.3 nm, x_{nref} = 22%).

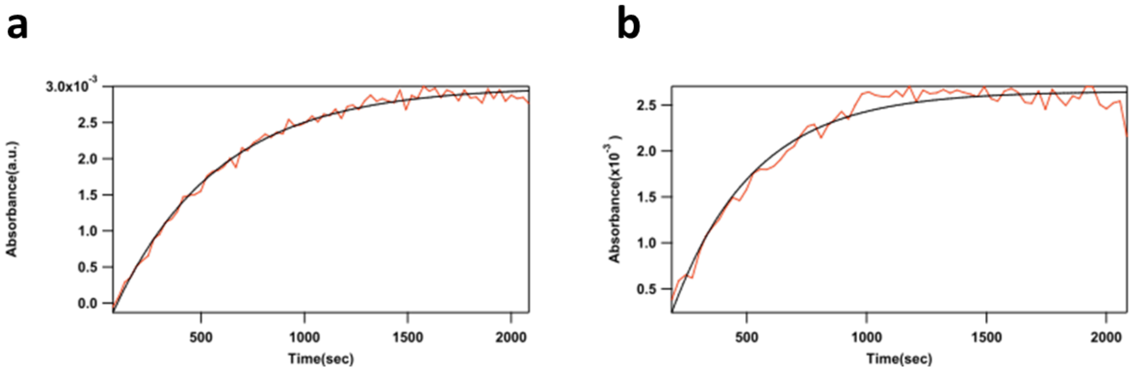

Figure S4. Reaction profile of Au:PVP-catalyzed 1-indanol oxidation. a, 1.3 nm Au:PVP (K-30) **b**, 7.0 nm Au:PVP (K-90)

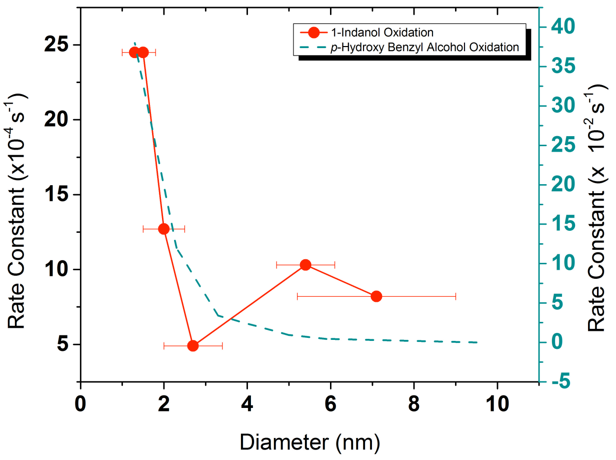

Figure S5. Reaction profile of Au:PVP (K-30)-catalyzed aerobic oxidation reaction. The data shows the relationship between rate constant and core size. The green dash line showed the reported size-dependency of Au:PVP (K-30) on aerobic oxidation of *p*-hydroxy benzyl alcohol. The red point show the size-dependency of Au:PVP (K-30) on aerobic oxidation of 1-indanol.

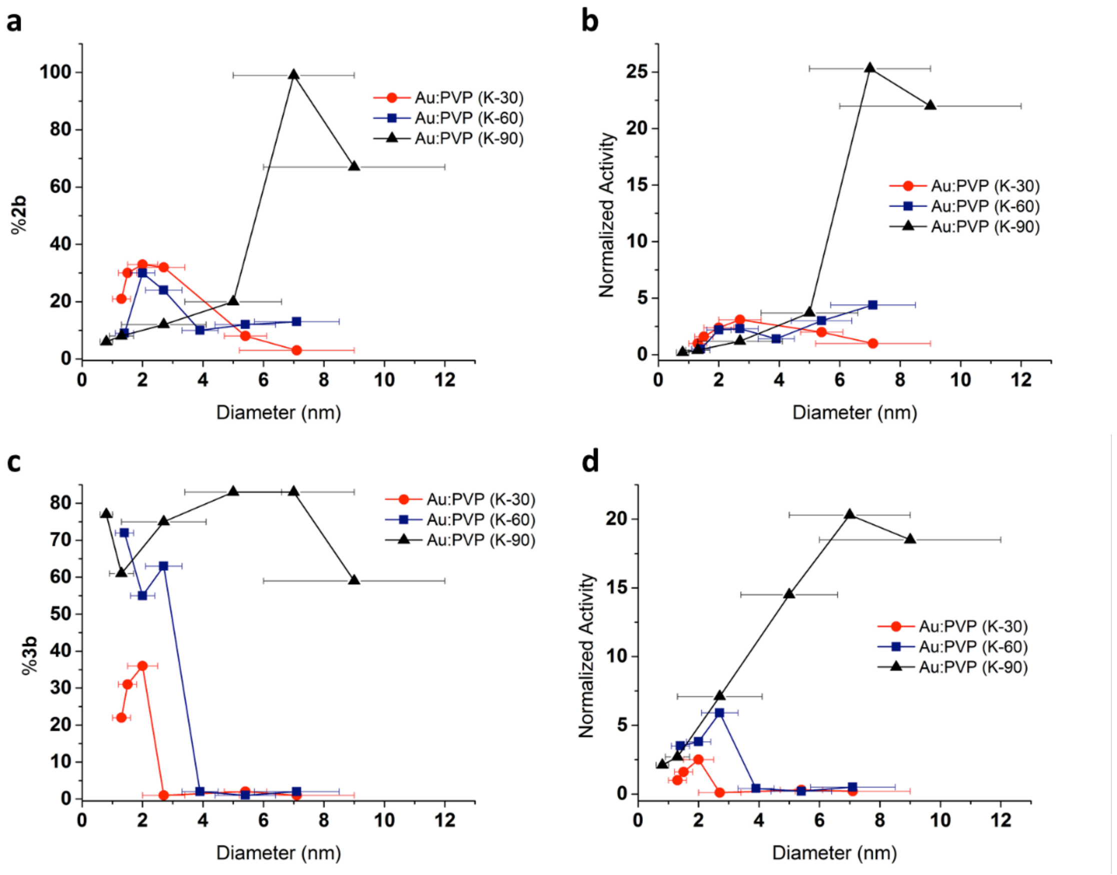

Figure S6. Size-dependency on oxidation reaction. The data shows the size-dependent properties of Au:PVP-catalyzed aerobic homocoupling reaction of $PhBF_3K$ (**a**,**b**) and intramolecular hydroalkoxylation (**c**,**d**). **a** and **c,** The relationship between %yield of desired product at 4 h of reaction time and core size. **b** and **d**, The relationship between normalized activity by using 1.3 nm Au:PVP (K-30) as standard and core size.

Table S4. The Electronic Structure Determination of Au:PVP

Entry	Au:PVP	Core size /nm	*CN* [a]	***R***/Å	***ΔE***/ eV	DW	Edge energy /eV	%Rf
1	PVP (K-30)	1.3±0.3	6.5±1.0	2.787±0.009	-2.656±1.847	0.086±0.011	11916.18[a]	1.5
2		1.5±0.3	7.0±0.4	2.781±0.010	-3.366±2.023	0.083±0.011	11916.06[a]	2.3
3		2.0±0.5	8.3±0.6	2.809±0.011	-2.455±1.995	0.900±0.005	11916.12[a]	1.2
4		2.7±0.7	8.8±0.6	2.817±0.011	-1.803±1.801	0.098±0.005	11916.18[a]	0.8
5		5.4±0.7	10.4±0.7	2.858±0.004	-2.602±0.809	0.073±0.005	11916.13[a]	0.4
6		7.1±0.7	10.6±1.7	2.866±0.009	-1.141±1.990	0.070±0.012	11916.13[a]	0.1
7	PVP (K-60)	1.4±0.3	7.8±0.5	2.810±0.011	-2.515±2.033	0.088±0.005	11916.12[a]	1.4
8		2.0±0.4	8.0±0.6	2.826±0.010	-1.660±2.016	0.087±0.005	11916.18[a]	1.4
9		2.7±0.6	8.4±0.6	2.829±0.004	1.222±0.754	0.092±0.005	11916.12[a]	2.4
10		3.9±0.6	8.5±1.3	2.858±0.009	-0.168±1.938	0.076±0.011	11916.13[a]	0.5
11		5.3±0.7	10.5±1.6	2.860±0.009	-0.534±1.949	0.078±0.011	11916.07[a]	0.3
12		7.2±0.7	10.7±0.7	2.876±0.008	-0.220±1.911	0.069±0.005	11916.08[a]	0.3
13	PVP (K-90)	0.8±0.2	6.0±0.9	2.773±0.009	-5.341±1.905	0.078±0.010	11920.14[b]	2.0
14		1.3±0.4	6.0±0.4	2.773±0.008	-3.523±1.834	0.076±0.004	11919.88[b]	1.6
15		2.7±1.4	8.0±1.3	2.786±0.010	-3.545±1.930	0.092±0.012	11920.06[b]	0.6
16		5.0±1.6	9.0±0.6	2.810±0.010	-2.440±1.935	0.091±0.005	11916.03[a]	0.8
17		7.0±2.0	9.1±0.6	2.805±0.004	-2.446±0.755	0.092±0.005	11916.08[a]	2.3
18		9.1±3.4	10.6±1.8	2.817±0.011	-1.564±1.874	0.099±0.012	11920.0[b]	0.9

CN = coordination number, *R* = distance, *ΔE* = energy difference in the absorption threshold between the Au foil and Au:PVPs, DW = Debye-Waller factor; [a,b] The edge energy of Au foil was 11916.3 and 11920.3 eV, respectively.

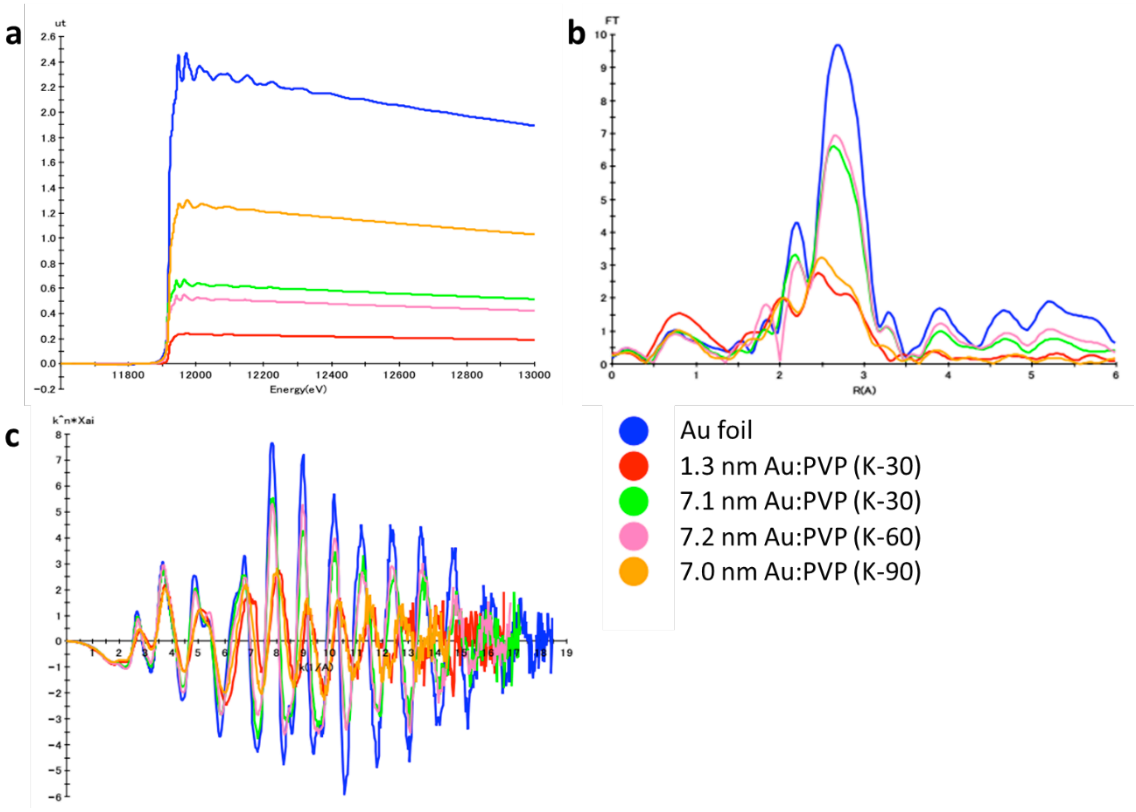

Figure S7. The representative XAS spectra. The data shows the XAS spectra of Au foil and AuPVPs at AuL_{III} edge. **a**, XANES spectra. **b**, AuL_{III} EXAFS in distance space. **c**, in *k* space

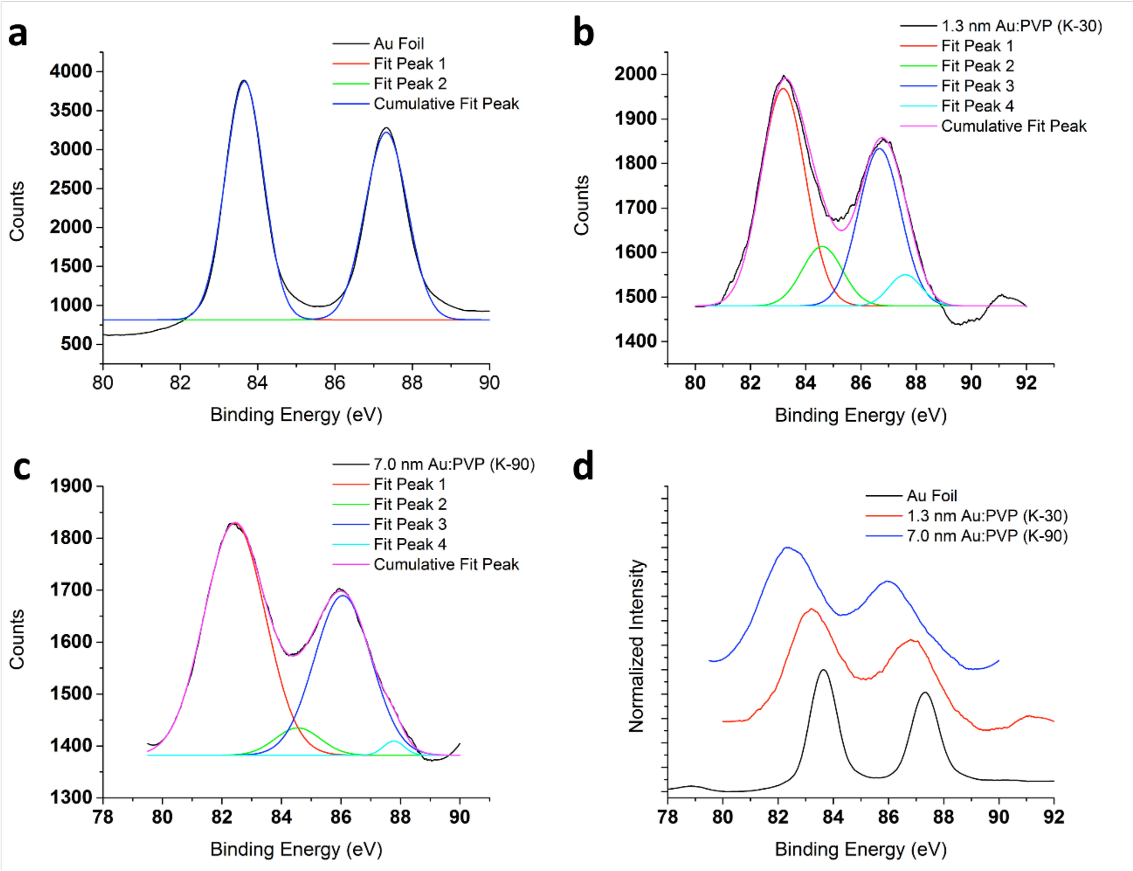

Figure S8. The representative XPS spectra. The data shows the XPS spectra of Au_{4f}. **a**, Au foil. **b**, 1.3 nm Au:PVP (K-30). **c**, 7.0 nm Au:PVP (K-90). **d**, The overlay XPS spectra of Au_{4f}.

Table S5. The colloid structure determination of Au:PVP

Entry	PVP	Core size (nm)	Colloid size (nm)
1	K-30	1.3±0.3	73±32
2		1.5±0.3	61±41
3		2.0±0.5	38±28
4		2.7±0.7	48±29
5		5.4±0.7	52±26
6		7.1±0.7	49±10
7	K-60	1.4±0.3	64±40
8		2.0±0.4	56±38
9		2.7±0.6	61±24
10		3.9±0.6	66±30
11		5.3±0.7	15±5
12		7.2±0.7	16±2
13	K-90	0.8±0.2	76±34
14		1.3±0.4	97±14
15		2.7±1.4	61±10
16		5.0±1.6	6±3
17		7.0±2.0	10±1
18		9.1±3.4	14±7

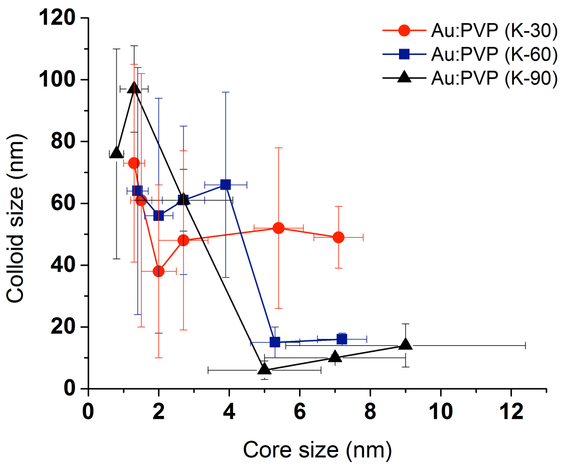

Figure S9. Colloid size of Au:PVP determined by IG method.

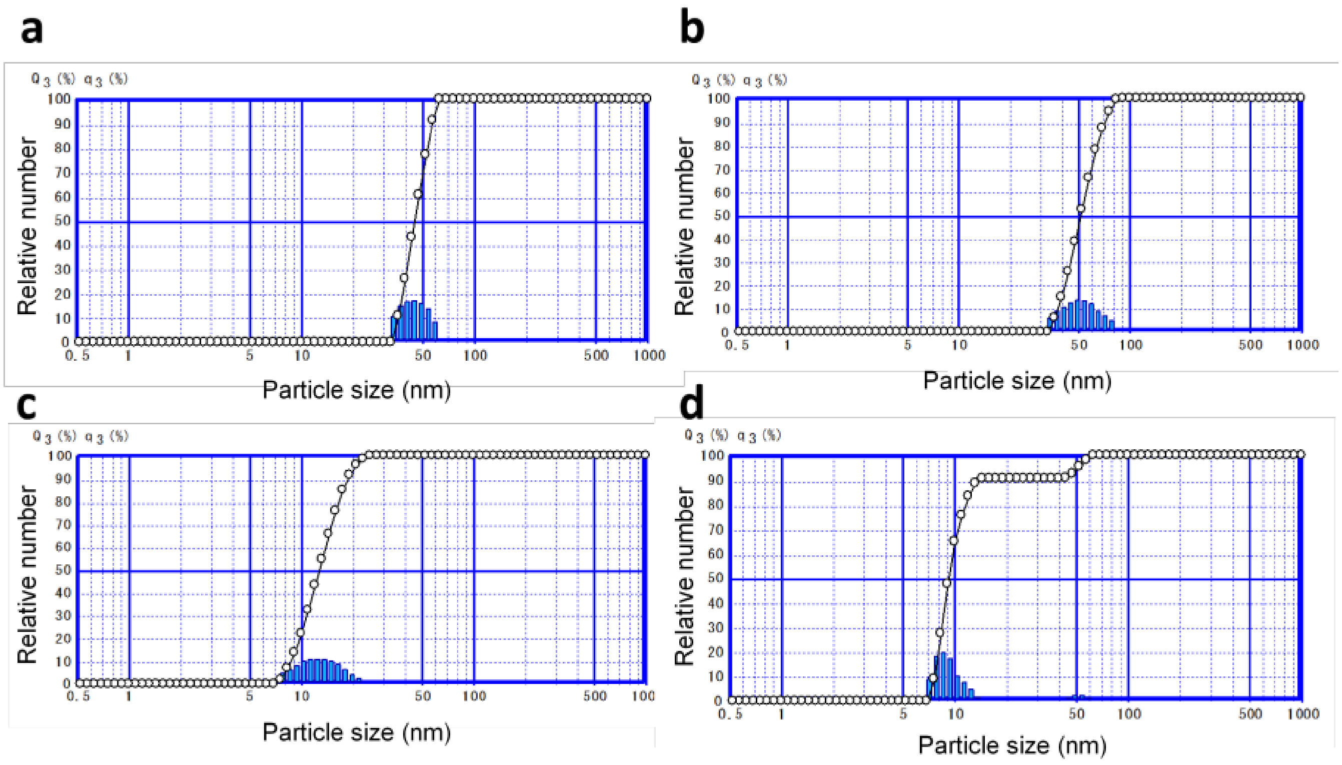

Figure S10. Size distribution of Au:PVP colloid determined by IG method. a, 1.3 nm Au:PVP (K-13). **b**, 7.1 nm Au:PVP (K-30). **c**, 7.2 nm Au:PVP (K-60). **d**, 7.0 nm Au:PVP (K-90).

6: References

1. Tsunoyama, H., Sakurai, H., Ichikuni, N., Negishi, Y. & Tsukuda, T. Colloidal gold nanoparticles as catalyst for carbon-carbon bond formation: Application to aerobic homocoupling of phenylboronic acid in water. *Langmuir* **20**, 11293-11296 (2004).

2. Haesuwannakij, S., Karuehanon, W., Mishra, V. L., Kitahara, H., Sakurai, H., Kanaoka, S. & Aoshima, S. Size-controlled preparation of gold nanoclusters stabilized by hydrophilic polymers with high viscosity using micro-flow reactor. *Monatsch. Chem.* **145**, 23-28 (2014).

3. Haesuwannakij, S., Poonsawat, T., Noikham, M., Sonsook, E., Yakiyama, Y., Dhital, R. N. & Sakurai, H. Size-controlled preparation of gold nanoclusters on hydroxyapatite through trans-deposition method. *J. Nanosci, Nanotechnol.*, *Advanced article* (2016) (DOI: 10.1166/jnn.2017.13777).

4. Tsunoyama, H., Sakurai, H. & Tsukuda, T. Size effect on catalysis of gold clusters dispersed in water for aerobic oxidation of alcohol. *Chem. Phys. Lett.* **429**, 528-532 (2006).

5. Chaki, N. K., Tsunoyama, H., Negishi, Y., Sakurai, H. & Tsukuda, T. Effect of Ag-doping on the catalytic activity of polymer-stabilized Au clusters in aerobic oxidation of alcohol. *J. Phys. Chem. C* **111**, 4885-4888 (2007).

6. Sakurai, H., Tsunoyama, H. & Tsukuda, T. Oxidative homo-coupling of potassium aryltrifluoroborates catalyzed by gold nanocluster under aerobic conditions. *J. Organomet. Chem.* **692***,* 368-374 (2007).

7. Vedejs, E., Chapman, R. W., Fields, S. C., Lin, S. & Schrimpf M. R. Conversion of arylboronic acids into potassium aryltrifluoroborates: Convenient precursors of arylboron difluoride Lewis acids *J. Org. Chem.* **60**, 3020-3027 (1995).

8. Kamiya, I., Tsunoyama, H., Tsukuda, T. & H. Sakurai, Lewis acid character of zero-valent gold nanoclusters under aerobic conditions: Intramolecular hydroalkoxylation of alkenes. *Chem. Lett.* **36**, 646-647 (2007).

9. Kitahara, H. & Sakurai, S. Catalytic activity of gold nanoclusters in intramolecular hydroamination of alkenes and alkynes with toluenesulfonamide under aerobic and basic conditions. *J. Organomet. Chem.* **696***,* 442-449 (2011).
